# Pharmacological Activities of *Zingiber officinale* Roscoe: Inhibition of HSA Protein Glycation, Structure Stability and Function Restoration

**DOI:** 10.3390/ph17111469

**Published:** 2024-11-01

**Authors:** Mohd Wajid Ali Khan, Subuhi Sherwani, Muna H. E. Alshammari, Abdulmohsen K. D. Alsukaibi, Wahid Ali Khan, Ashanul Haque, Khalaf M. Alenezi, Uzma Shahab

**Affiliations:** 1 Department of Chemistry, College of Sciences, University of Ha’il, Ha’il 55473, Saudi Arabia; moonaa243@gmail.com (M.H.E.A.); a.alsukaibi@uoh.edu.sa (A.K.D.A.); a.haque@uoh.edu.sa (A.H.); k.alenezi@uoh.edu.sa (K.M.A.); 2 Medical and Diagnostic Research Center, University of Ha’il, Ha’il 55473, Saudi Arabia; s.sherwani@uoh.edu.sa; 3 Department of Biology, College of Sciences, University of Ha’il, Ha’il 55473, Saudi Arabia; 4 Department of Clinical Biochemistry, College of Medicine, King Khalid University, Abha 62521, Saudi Arabia; wahidalikhan@rediffmail.com; 5 Department of Biochemistry, King George Medical University, Lucknow 226003, India; uzmashahab@gmail.com

**Keywords:** non-enzymatic glycation, natural products, inhibitors, HSA, AGEs, antiglycation, oxidative stress, antioxidation

## Abstract

Background: Controlled non-enzymatic glycation reactions are common under normal physiological conditions. However, during elevated blood glucose conditions, the glycation reactions are accelerated, leading to the formation of toxic compounds such as advanced glycation end products (AGEs). Several natural products are now being investigated as protective agents against glycation to preserve blood protein structure and functions. Methods: Human serum albumin (HSA) was glycated with 0.05 M α-D-glucose alone or in the presence of *Zingiber officinale* Roscoe (ginger) extract (0.781–100 μg/mL) for 10 weeks, and biochemical, biophysical, and computational analyses were carried out. Results: HSA glycated for 10 weeks (G-HSA-10W) resulted in significant production of ketoamines, carbonyl compounds, and AGE pentosidine. Notable structural alterations were observed in G-HSA-10W, ascertained by ultraviolet (UV), fluorescence, and circular dichroism (CD) studies. Antioxidant, anti-glycating, AGEs inhibitory, and antibacterial effects of ginger extracts were observed and attributed to the presence of various phytochemicals. Molecular docking studies suggested that the compounds 8-shagaol and gingerol exhibited strong and multiple interactions with HSA. Molecular simulation analysis suggests HSA attains a high degree of conformational stability with the compounds gingerol and 8-shogaol. Conclusions: These findings showed that ginger extract has an antioxidant function and can prevent glycation-induced biochemical and biophysical alterations in HSA. Thus, aqueous ginger extract can be utilized to combat glycation and AGE-related health issues, especially diabetes, neurological disorders, inflammatory diseases, etc.

## 1. Introduction

Human serum albumin (HSA) is the predominant protein in plasma or serum and performs a variety of functions. It has a unique ability to bind to a wide range of small biological and synthetic molecules, acting as a chemical sponge [1]. It is a well-known in vivo protective agent against free radicals and mitigates damage caused by reactive molecules [1]. In addition to improving drug solubility and decreasing toxicity, albumin binding prolongs the half-life of drugs [2]. HSA increases the solubility of hydrophobic drugs in plasma, allowing them to either be delivered to specific tissues and organs or be eliminated when they become toxic [3]. Therefore, a decline in albumin concentrations or compromise of its functionality can have major effects on tissue fluid distribution, metabolism, nutrition, and substrate transport, among others [4].

Glycation of proteins produces AGEs. These AGEs are produced by a complicated sequence of parallel and sequential interactions between nucleophilic groups on proteins and reducing sugars as well as other molecules [5]. In fact, AGE production outpaces blood glucose increase, indicating that even a small rise in diabetes blood glucose levels might lead to a significant increase in AGE accumulation [6]. One of the major concerns of the glycation process is the generation of high levels of reactive oxygen species (ROS). Glucose auto-oxidation can generate high levels of ROS [7]. The pathogenicity of glycation and the formation of AGEs is due to the production of ROS, cross-linkage, aggregation, protein misfolding, and precipitation of different proteins [8,9]. Protein misfolding and aggregation are implicated in numerous diseases, including familial amyloidosis, Alzheimer’s disease, neurodegenerative disorders, and pancreatic islet amyloidosis [9]. Additionally, glycation-induced biological products are known to be mainly associated with aging, diabetes and its complications (diabetic retinopathy, atherosclerosis, nephropathy), inflammatory changes, skin photoaging, osteoporosis, and progression of some tumors [10].

Exploring the potential of natural products to inhibit glycation of serum proteins may offer a strategy to reduce the incidence of these diseases and their associated complications. For centuries, ginger has been a key component of remedies for treating colds, nausea, migraines, hypertension, constipation, catarrh, neurological illnesses, stroke, gingivitis, rheumatism, toothache, asthma, and diabetes [11,12]. Ginger root of *Zingiber officinale* Roscoe is a nutrient-dense food, containing essential micronutrients including vitamin B6, vitamin C, potassium, magnesium, manganese, copper, and dietary fiber with substantial amounts of water. It includes about 18% carbohydrates, 2% protein, and 1% fat. It is also abundant in bioactive compounds, particularly phytochemicals and polyphenols, which contribute to its health-promoting properties [13]. Recent studies have shown that the phytochemicals present in ginger exhibit potent antioxidant, anti-inflammatory, and anti-apoptotic properties, both in vivo and in vitro [14]. Compounds present in ginger include gingerols, paradols, 3-dihydroshogaols, shogaols, 3-dihydroparadols, acetyl derivatives of gingerols, mono- and di-acetyl derivatives of gingerdiols, diarylheptanoids, 1-dehydrogingerdiones, and methyl ether [15,16]. Among these, gingerols, shogaols, and paradols are the three primary active components, all derived from terpenes, a class of organic compounds known for their therapeutic effects. These compounds are responsible for many of ginger’s anti-inflammatory and antioxidant benefits. The recommended daily intake of ginger powder typically falls between 170 mg and 1 g, offering a concentrated source of its beneficial components [13].

Studies show that aqueous extract of ginger contains saponins, flavonoids, amines, alkaloids and terpenoids [17]. Gingerol, shogaol, α-sitosterol, α-bisabolene, α-sesquiphellandrene, α-curcumene, and zingiberene are found in aqueous extracts of ginger [18,19]. In their findings, Manuhara et al. showed the presence of β-sesquiphellandrene; α-curcumene; and β-bisabolene in an aqueous extract of ginger [20]. However, the amounts of these compounds recovered in aqueous extracts were reportedly lower than those obtained through alcoholic extraction methods.

Based on the aforementioned studies, an aqueous extract of ginger was selected to mimic normal physiological conditions in vitro. Using water as a solvent ensures minimal interference, avoiding additional structural modifications. It was hypothesized to utilize the ginger aqueous extract in different glycation reactions to assess its efficacy in inhibiting glycation reactions as well as AGE formation.

## 2. Results

### 2.1. Biochemical Analysis

#### 2.1.1. Extraction and Phytochemical Screening

The total phenolic components in ginger extract were estimated to be 42.28 ± 0.33 mg of gallic acid equivalent/g of the extract weight. These findings indicated that the extract’s total flavonoid concentration was 21.28 ± 0.16 mg quercetin equivalents (QEs)/g weight.

#### 2.1.2. Antioxidant Study

**Figure 1:**
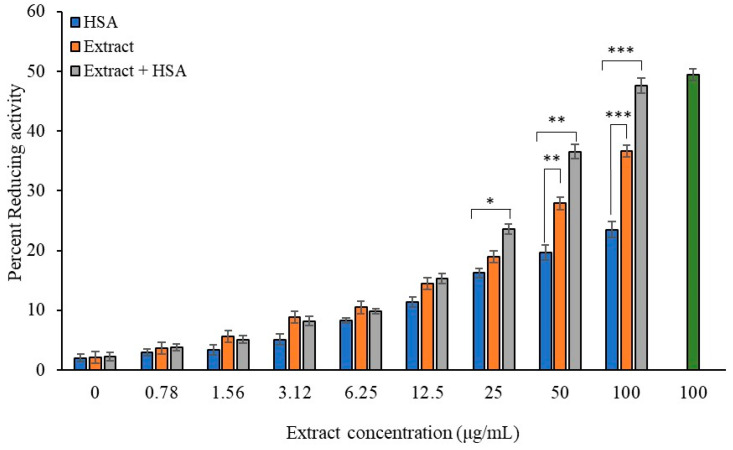
Radical scavenging activity of ginger extract for H_2_O_2_ in absence and presence of HSA. Ginger extracts alone (Blue column) with various concentrations (0, 0.78, 1.56, 3.12, 6.25, 12.5, 25, 50, and 100 µg/mL) or in presence of HSA (40 mg/mL) (orange column) were used in the reactions. Ascorbic acid (100 µg/mL) served as standard (green column). HSA (40 mg/mL) alone served as second control. Values are presented as mean ± SD values and *p* values represent significance. Significance is represented by the symbols *, **, and *** for *p* < 0.05, *p* < 0.01, and *p* < 0.001, respectively.

Hydrogen peroxide (H_2_O_2_) is a well-studied compound which can generate hydroxyl radicals, leading to oxidative damage of proteins or DNA. Ginger is known for its antioxidant activity and has the ability to reduce hydrogen peroxide (Figure 1). Compared to lesser ginger extract concentrations (0–25 μg/mL), 100 μg/mL of ginger extract eliminated H_2_O_2_ at the highest rate (36.63%; *p* < 0.001), exhibiting strongest efficacy. The extract’s high polyphenol concentration may be attributed to its high antioxidant activity. The natural antioxidant and effective redox buffer, ascorbic acid, can greatly reduce and neutralize reactive oxygen species. Hence, ascorbic acid was used in this assay as a positive control for antioxidants.

Furthermore, HSA has intrinsic antioxidant capacity based on its ligand binding ability, which needs to be evaluated in the presence of extract. In this assay, the physiological concentration of HSA (40 mg/mL) was taken. HSA alone or in combination with the extract (varying concentration, (0–100 µg/mL) was tested in an H_2_O_2_ radical reducing activity assay. The reducing activity was significantly enhanced when HSA was mixed with the extract as compared to just the extract or HSA alone. Notably, an extract concentration of 25 µg/mL or above combined with HSA showed a significant increase in H_2_O_2_ radical reducing activity. Moreover, extract concentrations of 50 µg/mL or above exhibited significantly greater reducing activity compared to HSA alone. These findings indicate that ginger extract enhances the antioxidant function of HSA. In contrast, G-HSA did not show appreciable H_2_O_2_ radical reducing activity.

### 2.2. Glycation Reaction Standardization

**Figure 2:**
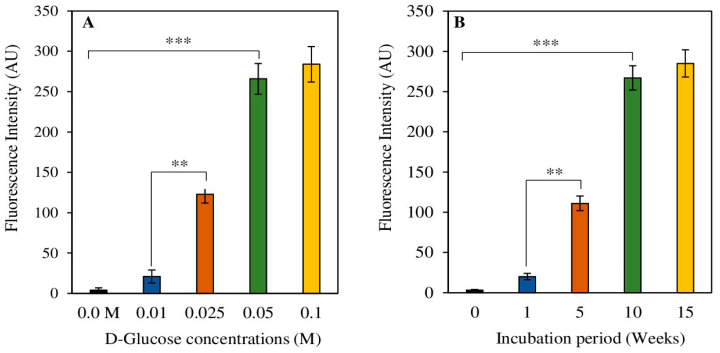
Standardization of glycation reaction of protein HSA. Varying concentrations of D-Glucose (0–0.1 M) (**A**) and different durations (0–15 weeks) (**B**) of incubation periods were used in the glycation reaction. Data shown in the figures are mean ± SD values and *p* values represent significance. Significance changes are represented by the symbols ** and *** which mean *p* < 0.01 and *p* < 0.001, respectively.

Comprehensive experimental setups were conducted to standardize the glycation of HSA with D-glucose under in vitro conditions. Varying concentrations of D-glucose were used to standardize the glycation of protein HSA. AGE pentosidine fluorescence intensities were estimated to optimize HSA glycation, as pentosidine is a well-known AGE molecule. A concentration of 0.05 M of α-D-glucose showed significantly higher formation (*p* < 0.001) levels of AGE molecules (Figure 2A). A concentration of 0.025 M of α-D-glucose also demonstrated increased levels (*p* < 0.01) of pentosidine formation as compared to the 0.01 M α-D-glucose concentration (Figure 2A).

Various incubation durations were tested to determine the optimal time for the glycation reaction, where 10 weeks of incubation showed a significantly (*p* < 0.001) higher amount of pentosidine formation as compared to 1-week and 5-week incubations (Figure 2B), respectively. An incubation period of 5 weeks also showed notably elevated (*p* < 0.01) pentosidine fluorescence as compared to the 1-week sample. There was no significant difference between fluorescence intensities between samples incubated for 10 weeks and those incubated for 15 weeks.

#### 2.2.1. Glycation Reaction Metabolite Detection

##### Ketoamines Synthesis

**Figure 3:**
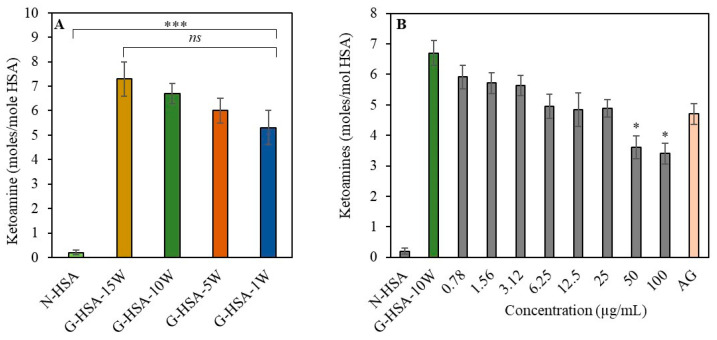
Effect of time and ginger concentration on formation of ketoamines. Ketoamines are produced during the glycation process with varying durations of incubation (1, 5, 10, and 15 weeks) (**A**). Inhibition of ketoamines formation with ginger extract (0.78–100 µg/mL) when incubated for 10 weeks with glycation systems (**B**). Aminoguanidine represented by AG. Other conditions were similar for all the reactions. Data shown in the figures are mean ± SD values and *p* values represent significance. Significance changes are represented by the symbols *, *** representing *p* < 0.05, *p* < 0.001, respectively; ns: not significant.

Early interactions between free amino groups, which contain some lysyl residues and D-glucose, may lead to the formation of ketoamines. Ketoamines are glycation products that form early in the glycation process. The nitro blue tetrazolium chloride (NBT) method was used for the colorimetric assessment of glycation-induced ketoamine generation in reaction samples. Ketoamines were found when protein HSA was treated with D-glucose (0.01 M) for varying durations of time (0–15 weeks). The generation of ketoamines was observed in all glycated samples of HSA [G-HSA-15W (7.3 ± 0.7), G-HSA-10W (6.7 ± 0.4), G-HSA-5W (6.0 ± 0.5), and G-HSA-1W (5.3 ± 0.7) moles/mol HSA] (Figure 3A). Additionally, significant differences (*p* < 0.001) were observed in the number of ketoamine moieties detected in glycated samples compared to non-glycated protein (0.20 ± 0.10 moles/mol HSA).

The production of ketoamines was examined in native HSA (N-HSA), G-HSA-10W, and G-HSA-10W samples with varying concentrations of ginger extracts. Extract concentrations (0–2.5 µg/mL) did not lead to notable differences in the ketoamine production (Figure 3B). A well-known synthetic antiglycating agent called aminoguanidine can inhibit the final stage of the glycation process [21]. Similarly, our finding also showed no significant inhibition in the formation of ketoamine with the addition of aminoguanidine. However, higher extract concentrations (50–100 µg/mL) showed significant (*p* < 0.05) ketoamine inhibition. This result shows that substantial amounts of ginger extract are required to achieve a notable reduction in the formation of ketoamine.

##### Detection of Protein-Bound Carbonyl Compounds

**Figure 4:**
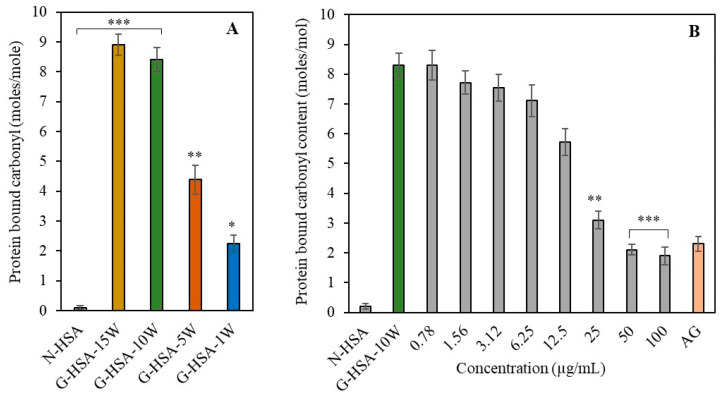
Effect of ginger extract and incubation time on carbonyl compound formation in HSA. Carbonyl compounds are formed during the glycation process under different durations of incubation (1, 5, 10, and 15 weeks) (**A**). Inhibition of carbonyl compound formation with extract and varying ginger extract concentrations (0.78–100 µg/mL) were incubated with glycation systems for 10 weeks (**B**). Other conditions were similar for all the reactions. Data shown in the figures are mean ± SD values. Significance changes are represented by the symbols *, **, *** which mean *p* < 0.05, *p* < 0.01, *p* < 0.001, respectively.

The quantified carbonyl compounds associated with the HSA molecules were regarded as a general indicator of protein oxidation, potentially leading to the addition of carbonyl groups to amino acid residues [22]. Carbonyl groups linked to proteins were found to vary depending on the period of incubation [G-HSA-15W (8.9 ± 0.35), G-HSA-10W (8.4 ± 0.40), G-HSA-5W (4.38 ± 0.49), and G-HSA-1W (2.4 ± 0.49) moles/mol HSA] (Figure 4A). This result showed that significantly higher levels (*p* < 0.001) of protein-bound carbonyl compounds were generated in the glycation reaction of HSA with glucose when incubated for 10–15 weeks. The G-HSA-5W and G-HSA-1W samples also showed significant changes (*p* < 0.01 and *p* < 0.05, respectively) compared to the N-HSA sample (0.02 ± 0.01 moles/mole of HSA).

No significant difference was observed in the generation of carbonyl compounds between the G-HSA-15W and G-HSA-10W samples. Therefore, the G-HSA-10W sample was used for the inhibition assays. The generation of carbonyl content in G-HSA-10W was shown to significantly decrease when treated with ginger extract concentrations of 25, 50, and 100 μg/mL as compared to the G-HSA-10W sample without the inhibitor. It was discovered that ginger extract at a dose of 50 μg/mL (*p* < 0.001) was most efficient at lowering the development of carbonyl content during the glycation processes. Further increases in ginger concentration showed a minimal additional effect on carbonyl content (Figure 4B).

### 2.3. Toxicity of Ginger Extract

**Figure 5:**
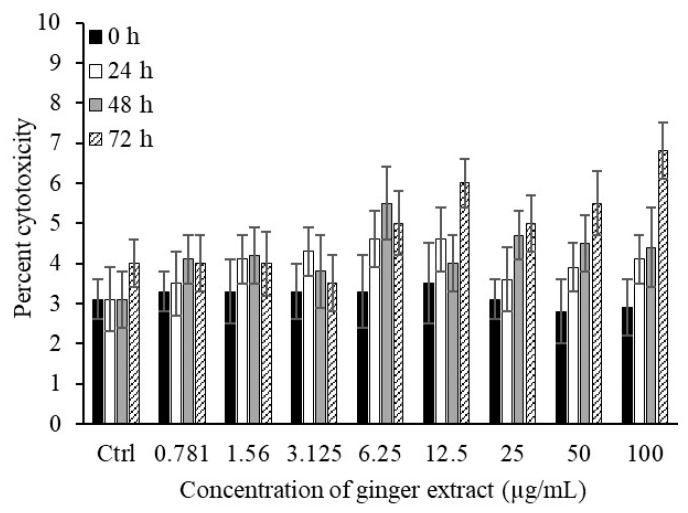
Treatment of human PBMCs with ginger extract. Ginger extract of varying concentrations (0.781–100 µg/mL) was added to human PBMCs (1 × 10^4^ cells/mL) for different durations of time (0–72 h). Data shown in the figures are mean ± SD values.

Prior to utilizing ginger extract for human applications, it is essential to evaluate its effects on normal human cells. The results indicate that ginger extract (0.781–100 µg/mL) is non-toxic to human blood cells. In a 3-[4,5-dimethylthiazol-2-yl]-2,5-diphenyltratrazolium bromide (MTT) assay, freshly isolated peripheral blood mononuclear cells (PBMCs) were used to determine the toxicity of the ginger extract (Figure 5). Across all tested concentrations (0.781–100 µg/mL), only minimal cytotoxicity (3–5.1%) was observed. Even at higher concentrations (>100 µg/mL), no significant PBMC mortality was detected. Control samples of PBMCs were cultured for the same amount of time without exposure to ginger extracts (0, 24, 48, and 72 h). This outcome demonstrated that ginger extract is not harmful to human blood cells.

### 2.4. Antibacterial Assay

To test the antibacterial ginger aqueous extract activity with HSA, the antibacterial assay was performed with extract alone or in the presence of has, as presented in Table 1. For comparative purposes, the outcomes of tetracycline activity are presented in Table 1.

The antibacterial efficacy of the examined extract was assessed by determining minimum inhibitory concentrations against seven species of both Gram-positive and Gram-negative bacteria. The MIC values for ginger extract ranged from 0.388 mg/mL to 41 mg/mL, while the MIC values for a combination of the extract and HSA ranged from 0.336 mg/mL to 43 mg/mL. The degree of antibacterial activity exhibited variability based on the bacterial species involved. It was evident that in the presence of HSA with ginger extract, all the tested bacteria (except *Staphylococcus aureus*) showed decreased MICs as compared to the extract alone. Significant enhancements in the antibacterial activities of extract combined with HSA were found against *Pseudomonas aeruginosa*, *Bacillus subtilis*, and *Mycobacterium tuberculosis* when compared with the extract alone.

The extract exhibited moderate to potent antibacterial activity, displaying more pronounced inhibitory effects against Gram-positive compared to Gram-negative bacteria. The findings revealed that the *B. subtilis* standard strain was the most susceptible, with an MIC of 0.388 mg/mL and a minimum bactericidal concentration (MBC) of 0.336 mg/mL.

### 2.5. Biophysical Analysis

#### 2.5.1. Generation of AGE Pentosidine

**Figure 6:**
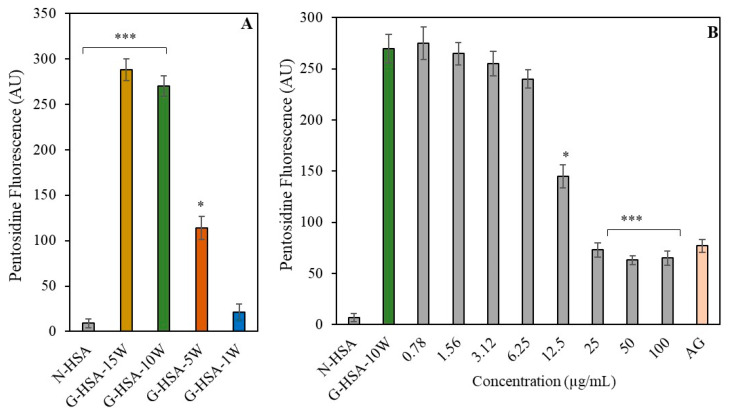
AGE pentosidine-specific fluorophores were analyzed in non-glycated and glycated HSA samples (**A**). Inhibition in the formation of pentosidine due to ginger extract (0.78–100 µg/mL) was determined in glycation of G-HSA-10W (**B**). Data shown in the figures are mean ± SD values. Significance changes are represented by the symbols *, *** which mean *p* < 0.05, *p* < 0.001, respectively.

The presence of the fluorophore pentosidine was evaluated in both modified HSA and unmodified HSA samples. When compared to N-HSA, the pentosidine-specific fluorescence in G-HSA-15W and G-HSA-10W was significantly higher (*p* < 0.001) (Figure 6A). High pentosidine fluorescence was also observed in HSA samples that had been glycated for five weeks (G-HSA-5W) (*p* < 0.05). But merely a week of glycation of the HSA sample (G-HSA-1W) resulted in decreased pentosidine fluorescence.

There was no significant difference in pentosidine fluorescence between the G-HSA-15W and G-HSA-10W samples. Thus, the G-HSA-10W sample was used in the inhibition assay. For 12.5 µg/mL of ginger extract, a notable (*p* < 0.05) reduction in AGE pentosidine production was evident in the reaction mixture. Additionally, extracts with higher doses (25, 50, and 100 µg/mL) significantly inhibited the production of pentosidine (*p* < 0.001) (Figure 6B).

#### 2.5.2. UV and Tryptophan Spectral Analysis

**Figure 7:**
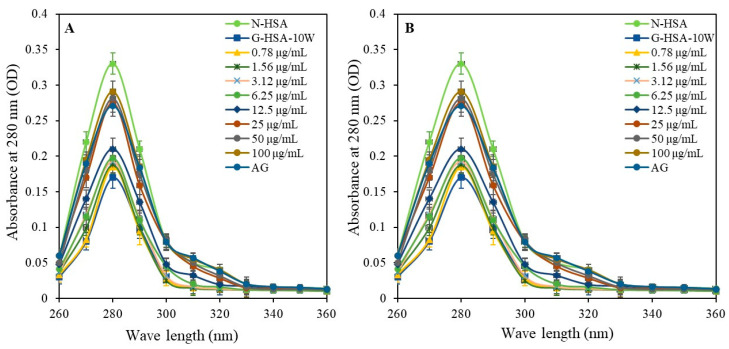
UV spectroscopic analysis was performed for N-HSA, G-HSA-1W, G-HSA-5W, G-HSA-10W, and G-HSA-15W (**A**). Effect of ginger extract on UV spectra of glycation of G-HSA-10W with different concentrations (0.78–100 µg/mL) (**B**). All recorded measurements were within the spectral range of 260 to 360 nanometers. Data shown in the figures are mean ± SD values.

Structural alterations of the HSA molecules during the glycation process were analyzed by UV and fluorescence analysis. The UV spectral characterization of samples for glycated and non-glycated protein molecules was performed and optical density (OD) at 280 nm was determined using a spectrophotometer. The G-HSA-15W sample showed the lowest UV intensities (0.17 ± 0.013; *p* < 0.001), followed by the G-HSA-10W (0.19 ± 0.014; *p* < 0.001), G-HSA-5W (0.26 ± 0.015; *p* < 0.05), and G-HSA-1W (0.30 ± 0.014; *p* = ns) samples (Figure 7A). There was no significant difference between N-HSA and G-HSA-1W. Increased hypochromicity in UV intensity suggested protein folding following incubation for 10 and 15 weeks during the glycation process.

The G-HSA-10W sample was used for inhibition studies to assess the UV intensities with varying concentrations of ginger extract as inhibitors. The addition of ginger extracts to the reaction mixture resulted in an increase in UV intensity, matching that of N-HSA. Notably, the UV intensity significantly increased with 25 μg/mL of ginger extract. This effect became more pronounced at higher extract concentrations (50 and 100 μg/mL) (Figure 7B). Therefore, ginger extract (25 µg/mL or more) reduces the protein folding of HSA molecules and helps to maintain the integrity of the original protein structure, which is otherwise altered by the glycation process.

**Figure 8:**
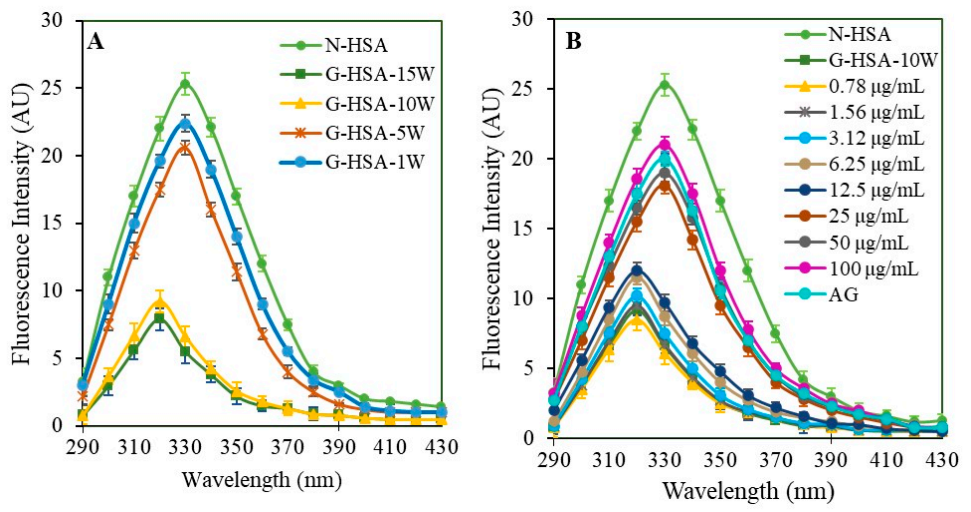
Tryptophan fluorescence emission spectra. Fluorescence analysis was performed for tryptophan molecules in N-HSA, G-HSA-1W, G-HSA-5W, G-HSA-10W, and G-HSA-15W (**A**) samples. Change in the tryptophan microenvironment during glycation of HSA (10 weeks) with different concentrations (0.78–100 µg/mL) (**B**). All the samples were excited with a wavelength of 285 nm. Data shown in the figures are mean ± SD values.

A unique feature of the HSA protein molecule is that it has a single tryptophan residue. Tryptophan residue fluorescence analysis demonstrated glycation-induced site-specific changes. Samples underwent analysis for tryptophan-specific fluorescence emission spectra within the 290 to 430 nm range, with an excitation wavelength of 285 nm. N-HSA (25.3 AU), G-HSA-1W (22.4 AU), and G-HSA-5W (20.6 AU) displayed emission spectra with a peak intensity at 330 nm. In contrast, the G-HSA-15W and G-HSA-10W samples exhibited intensities of 9.2 AU and 7.9 AU, respectively, at 320 nm (Figure 8A). The emission spectra showed a 10 nm blue shift due to glycation, and the fluorescence intensity significantly changed (N-HSA versus G-HSA-15W). The microenvironment of tryptophan residues in proteins may undergo changes due to glycation, resulting in a shift toward a more nonpolar environment, as the protein folds.

The glycation samples G-HSA-15W and G-HSA-10W showed no significant changes. Consequently, the G-HSA-10W sample was selected for the tryptophan-specific fluorescence intensity inhibition assay, with varying concentrations of ginger extracts. The study aimed to use different concentrations of the extract to block potential changes to the tryptophan microenvironment. A significant (*p* < 0.01) reversal of tryptophan fluorescence (18.1 AU) was evident in glycated samples when 25 μg/mL ginger extract was added to the reaction mixture. This effect was further significantly (*p* < 0.001) enhanced with higher concentrations (50 μg/mL and 100 μg/mL) of ginger extracts (8.7 AU) (Figure 8B). Additionally, at concentrations of 25, 50, and 100 μg/mL, there was a noticeable shift of 10 nm (blue shift) compared to the native protein. This result shows that a 25 g/mL or greater concentration of ginger extract induced exposure of the tryptophan residue to a polar environment and resistance to tryptophan microenvironment changes caused by glycation.

#### 2.5.3. Circular Dichroism

**Figure 9:**
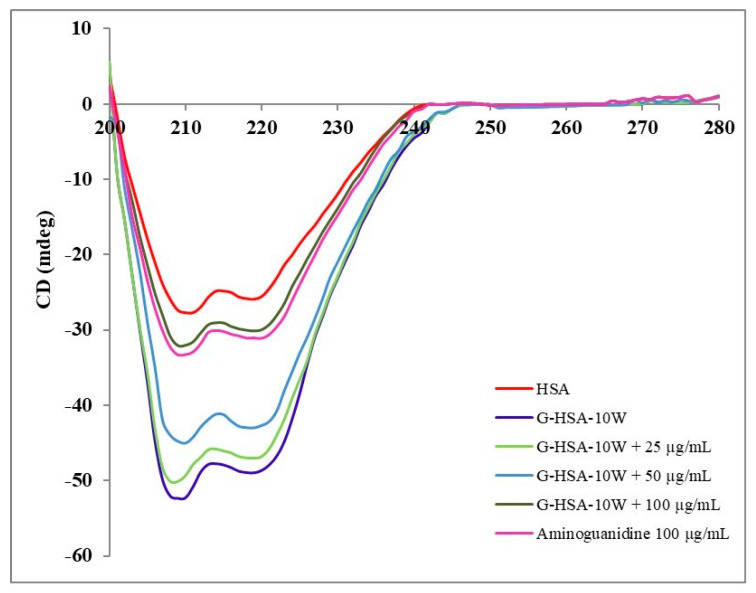
Secondary structural perturbation analysis of N-HSA and G-HSA-10W. Glycation reactions were mixed with ginger extract (25, 50, and 100 µg/mL). Aminoguanidine (µg/mL) served as positive glycation reaction inhibitor.

It is well known that glycation induces secondary structure alterations (α-helix, β-sheet, β-turn, and random coil), as also observed in this analysis (Table 2). CD analysis of G-HSA-10W indicated a significant decrease (−19.96%) in the α-helix structure but a substantial increase (+25.78%) in the β-sheet structure (Table 2). HSA glycation for 10 weeks also showed changes in the β-turn and random coil. As shown in Figure 9, the β structure changes due to the transition from native to glycated HSA, leading to an increase in protein misfolding or protein aggregation, which may contribute to structural perturbations associated with protein toxicity and dysfunction [23]. The introduction of ginger extract at various concentrations (25, 50, and 100 µg/mL) produced noticeable changes in the secondary structures, as observed graphically (Figure 9).

A reverse in the α-helix and β-sheet structure changes was observed with 12.5 µg/mL of ginger extract. A notable inhibition (*p* < 0.05) in β-sheet structural change was observed with 25 µg/mL of extract. A significant inhibition (*p* < 0.05) in α-helix was observed with 50 µg/mL of extract.

A concentration of 100 µg/mL of ginger extract showed maximum inhibition in all the secondary structure elements, including α-helix, β-sheet, β-turn, and random coil. α-helix structure percent change was inhibited between −19.96% and −6.05% (*p* < 0.001), β-sheet from +25.78% to +4.17% (*p* < 0.001), β-turn from +5.13% to 0% (*p* < 0.0001), and random coil from −5.21 to −3.48% (Table 2).

Random coil showed minimal effects which were non-significant. These findings revealed that ginger extract at 100 µg/mL exerts significant inhibition of secondary structure alterations of HSA when added to the glycation reaction with α-D-glucose (0.05 M) for 10 weeks. Thus, ginger extract showed a protective effect on HSA against glucose-induced glycation, which is clearly evident under abnormal hyperglycemic conditions (diabetes). Hence, ginger extract supports the preservation of normal structure and function of HSA, which is vital for several physiological functions.

### 2.6. Protection Against Protein Structure Disruption and Misfolding/Aggregation

#### 2.6.1. Decrease in Browning Intensity of G-HSA

Structural disruption of protein is induced by a variety of chemical agents and one of these is the non-enzymatic glycation of protein molecules [7]. In this experiment, the presence of ginger extract in glycated samples showed significant inhibition of the browning intensity at concentrations of 50 µg/mL (28%; *p* < 0.01) and 100 µg/mL (43%; *p* < 0.001), as compared to the glycated sample without extract.

#### 2.6.2. Inhibition of Protein Misfolding or Aggregation Percent

Non-enzymatic glycation of macromolecules, such as proteins, causes secondary structural changes, as evidenced by CD analysis. Secondary structure analysis revealed the formation of a β-sheet structure, which is involved in the formation of protein aggregates or misfolding of protein. When the glycation reaction of serum albumin was carried out in the presence of ginger extract, a significant inhibition in protein aggregation was observed. The reduction in aggregation was evident at concentrations of 25 µg/mL (21%; *p* < 0.05), 50 µg/mL (32%; *p* < 0.01), and 100 µg/mL (57%; *p* < 0.001), as compared to the glycated control sample without extract.

These findings clearly indicate the protective function of ginger against protein structure disruption, protein stability enhancement, reduction in protein misfolding or aggregation, and eventually antiglycation of blood protein albumin. These attributes highlight the valuable and important biological effects of ginger.

### 2.7. Computational Studies

#### Molecular Docking for Receptor and Ligand Studies

The aqueous extract of ginger contains various phytochemicals known for eliciting bioactivities [24]. We selected the compounds (α-curcumene, β-sesquiphellandrene, zingiberen, gingerol, 8-shogaol, α-sitosterol, and α-bisabolene) for in silico studies, based on the available literature [17,18,19,20]. The concentrations of these compounds are reportedly less in aqueous extracts as compared to those obtained through alcoholic extraction methods. Studies have shown that aqueous extract of ginger contains saponins, flavonoids, amines, alkaloids, and terpenoids [17]. Specific compounds identified in aqueous extract of ginger include gingerol, shogaol, α-sitosterol, α-bisabolene, α-sesquiphellandrene, α-curcumene, and zingiberene [18,19]. Manuhara et al. reported the presence of β-sesquiphellandrene; α-curcumene; and β-bisabolene in an aqueous extract of ginger [20].

**Figure 10:**
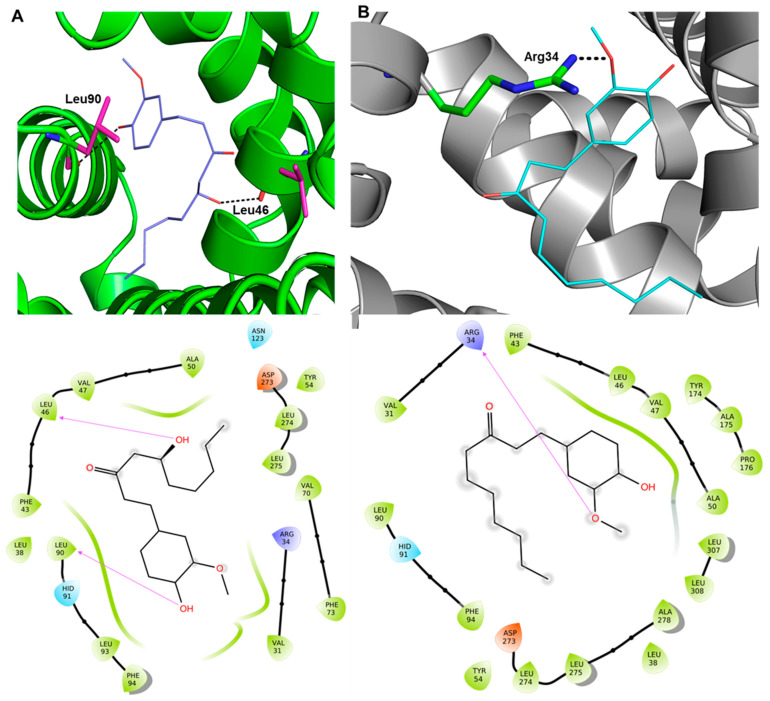
Three-dimensional and two-dimensional docking interactions between HSA, compound **4** (**A**), and compound **5** (**B**).

The possible participation of these aqueous extract compounds in inhibiting glycation-induced biochemical and biophysical alterations in HSA was evaluated by molecular docking. The molecular docking was performed between the HSA protein (PDB: 7ov1) and compounds **1**–**7** (α-curcumene, β-sesquiphellandrene, zingiberen, gingerol, 8-shogaol, α-sitosterol, α-bisabolene). The outcome of the study is depicted in Figure 10 and Table 3. As expected, compounds (**4**) gingerol and (**5**) 8-shogaol had the most favorable results, which have been reported to be present in significant quantities [24].

Compounds **4** and **5** interacted with protein HSA via conventional and non-conventional interactions. For example, compound **4** interacted with Leu46 and Leu90 through the hydrogen bond while residues like Val31, Arg34, Leu38, Phe43, Val47, Ala50, Tyr54, Val70, Phe73, His91, Leu93, Phe94, Asn123, Asp273, Leu274, and Leu275 were involved in hydrophobic, van der Walls, and other interactions. Compound **5**, which is the reduced form of compound **4**, formed an H-bond with Arg34 within a 2.5 Å distance. On the other hand, Val31, Leu38, Phe43, Leu46, Val47, Ala50, Tyr54, Leu90, His91, Phe94, Asp273, Leu274, Leu275, Ala278, Leu307, and Leu30 were also found to interact with compound **5**. Since only compounds **4** and **5** were able to interact with HSA via strong multiple interactions with higher binding affinities (>−7 kcal/mol), they were selected for molecular dynamics (MD) simulation studies (vide-supra).

**Figure 11:**
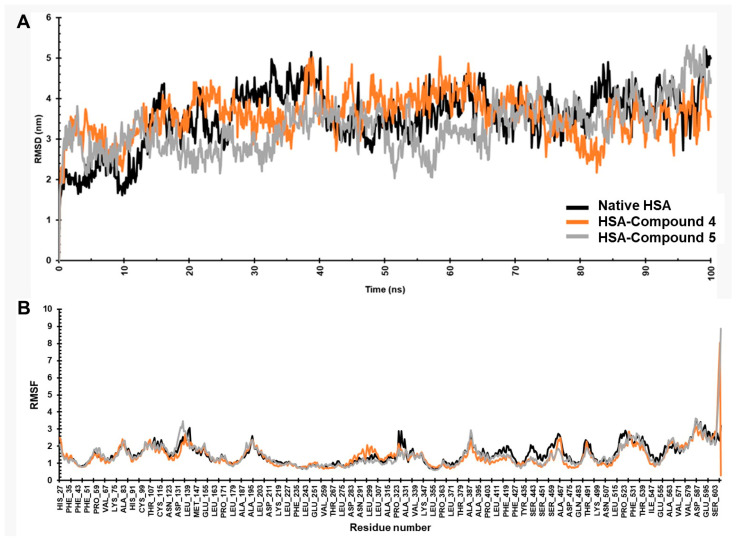
MD simulation results. (**A**) RMSD and (**B**) RMSF plots of native HSA and complexes.

MD simulations of HSA in its native state and complexed with compounds **4** and **5** were conducted at the 100 ns timescale. To ensure physiological relevance, simulation was carried out in a zero-net charge environment, with the addition of Na^+^ and Cl^−^ ions surrounded by water molecules (concentration of 0.15 M). The simulation box for HSA and the complex was maintained at a temperature of 300 K. The results were analyzed by monitoring root mean square deviation (RMSD, Figure 11A) and root mean square fluctuation (RMSF, Figure 11B). The native form of HSA reached equilibrium after 15 ns (Figure 11A), exhibiting a stable conformation with an RMSD ~3.54 nm, which is acceptable for native protein. The complex formed by compound **4** with HSA demonstrated higher stability, with RMSD plateauing after 20 ns and converging at an average value of 3.53 nm (Figure 11B). Contrary to this, the HSA–compound **5** complex exhibited an average RMSD of 3.28 nm, the lowest value among the studied complexes.

The root mean square fluctuation (RMSF) plot of native HSA indicates notable oscillations (RMSD > 1.54 nm) in the N-terminal and C-terminal residues. However, the trajectory was remarkably consistent throughout the simulation when complexed with compounds **4** and **5** (RMSF < 1.42 nm). This suggests a high degree of conformational stability induced by the compounds. We noted that the total number of atoms in the simulated system was higher in the case of compound **5**, which explains its higher RMSF value. Further RMSF analysis of complex containing compound **5** (Figure 11B) revealed relatively lower fluctuations in residues 123 to 131, which did not interact with the compounds (as noted in the docking studies).

## 3. Discussion

Several biomolecules (proteins, lipids and nucleic acids) undergo the glycation process, which causes the formation of AGEs and the production of oxidative stress [25]. Glycation has a key role in several oxidative stress-related chronic diseases [24,26]. AGEs, carbonyl compounds, and ketoamines are just a few chemical products and intermediates produced when glucose and other sugars interact with biomolecules. These reactions can be accelerated due to hyperglycemia, aging, depression, and other chronic conditions [7,27].

Various human diseases have been treated using natural products and their constituents as they are considered non-toxic and safe. Survey data from the World Health Organization (WHO) in 2022 revealed that over 80% of individuals rely on traditional plant-based medications for primary healthcare [27]. Historically, it has been thought that natural products and their metabolites might be sources of therapeutically useful biomolecules used in drug development.

The family *Zingiberaceae* includes the perennial plant ginger (*Z. officinale*). As a medicinal plant, it stands out as highly efficacious and has been employed for centuries in herbal therapy for the prevention and treatment of diverse illnesses, including diabetes, hypertension, hyperlipidemia, atherosclerosis, and cardiovascular disease [28]. As one of the most potent natural antioxidants, ginger has been a staple in human diets for millennia. Studies have demonstrated that ginger extract contains significant amounts of phenolic and flavonoid compounds [15], as confirmed by the results of this study, contributing to its potent antioxidant and anti-inflammatory properties.

At least 14 bioactive compounds are highly prevalent in ginger, including [4]-gingerol, [6]-gingerol, [8]-gingerol, and [10]-gingerol, along with [6]-paradol, [14]-shogaol, [6]-shogaol, and other compounds like 1-dehydro-[10]-gingerdione, [10]-gingerdione, hexahydrocurcumin, and tetrahydrocurcumin. Additional active molecules include gingerenone A, 1,7-bis-(4′-hydroxyl-3′-methoxyphenyl)-5-methoxyhepthan-3-one, and methoxy-[10]-gingerol. These compounds are believed to contribute to ginger’s significant therapeutic potential [29].

Racemised amino acids, formation of dicarbonyl and AGEs, lipid peroxidation products, oxidative modifications of amino acids, and AGE-RAGE interactions are a few identifiable examples of the chemical harm caused by glycation [12,30,31]. Aging, inflammation, cancer, diabetes, cardiovascular disease, arthritis, cataracts, muscle degeneration, and delayed wound healing are a few note-worthy diseases linked to oxidative stress and ROS [32,33]. Furthermore, AGE formation is also associated with oxidative stress, which can potentially damage blood proteins. This study demonstrates that ginger extract exhibits the ability to lower H_2_O_2_ activity. Ginger contains a high level of total antioxidants surpassed only by pomegranate and berries [34].

All the glycated HSA samples showed high concentrations of ketoamines, proving that incubating HSA with D-glucose produces ketoamines. The highest quantity of ketoamines was generated in the G-HSA-15W sample, demonstrating the substantial impact of incubation time on the extent of glycation. This observation corresponds with findings from other studies [7], establishing a correlation between prolonged incubation periods and elevated glycation levels. Nonetheless, the presence of ginger extract resulted in a reduction in ketoamines, with a notable decrease observed at higher concentrations (50 and 100 μg/mL).

The concentration of carbonyl groups in a protein is considered an indicator of protein oxidation. During the glycation process, a series of reactions leads to the formation of AGEs, with carbonyl compounds being generated as intermediate products [7]. The inclusion of ginger extract at a concentration of 25 μg/mL or above significantly decreased the carbonyl content present in the glycated HSA sample. This finding provides even more evidence for the antioxidant properties of ginger extract components. The results show a decreased quantity of glycated carbonyl protein in the presence of ginger extract. This finding has been supported by the previous antioxidant studies conducted on ginger. A previous study conducted by Uz et al., 2009, showed that oral administration of ginger resulted in a significant reduction in the levels of tissue malondialdehyde, nitric oxide (NO), and protein carbonyl contents in the ischemia/reperfusion group of rats compared with the ischemia/reperfusion group of rats without ginger administration. Ginger supplementation in the diet resulted in a higher total antioxidant capacity and lower total oxidant status [35]. Nitric oxide is synthesized by inducible nitric oxide synthase (iNOS), an enzyme activated in response to various physiological stresses, such as inflammation. Overproduction of NO through iNOS can contribute to inflammatory conditions. Studies have shown that [6]-gingerol, a major bioactive compound in ginger, can effectively inhibit NO production in a dose-dependent manner. Specifically, [6]-gingerol was found to reduce the levels of iNOS in lipopolysaccharide (LPS)-stimulated mouse macrophages, a common model for studying inflammation. This suggests that [6]-gingerol exerts anti-inflammatory effects by modulating the NO pathway, providing potential therapeutic benefits for conditions associated with excessive NO production [36].

Toxicity analysis of the ginger extract is essential for its use as medicine. Thus, ginger extract was incubated with human PBMCs for different periods. Even at a greater extract concentration (100 µg/mL), no significant cell death was observed in freshly received PBMCs. This finding has been supported by a study that included human subjects where participants were administered oral doses of ginger ranging from 100 mg to 2 g, with blood samples collected over a period of 15 min to 72 h after a single dose. The analysis revealed that the free forms of key ginger bioactive compounds, including [6]-, [8]-, and [10]-gingerols, as well as [6]-shogaol, were not detectable in the blood. However, their respective glucuronide conjugates were present, indicating that these compounds are rapidly absorbed following oral consumption. The detection of these glucuronide conjugates suggests that gingerols and shogaol undergo extensive first-pass metabolism, being converted into more water-soluble forms, which facilitate their excretion and bioavailability. This underscores the efficient absorption and metabolic processing of ginger’s active components in the human body [37].

Pentosidine is a luminous and well-known AGE compound. Its levels in blood or tissue have been typically measured using HPLC or GC/MS [38]. However, these methods are often expensive, and the test material requires several pretreatment steps for acid hydrolysis and protein reduction. Consequently, specific fluorescence techniques have been employed to estimate pentosidine levels in blood [39,40], urine [40,41], tissue samples [42], and food items [43]. The glycated materials used in the current study exhibited strong fluorescence intensities associated with pentosidine. In addition, compared to native HSA, G-HSA-15W was shown to have the maximum fluorescence intensity. Conversely, ginger extract provided a safeguard against pentosidine production, with protective additive effects as the concentration of ginger extract was elevated. Hence, the findings illustrate that ginger extract provides protection to HSA from gluco-oxidation and cross-linking, induced by glycation.

HSA undergoes significant structural changes as a result of glycation, which decreases absorbance at 280 nm or hypochromicity. The observed effects experienced a notable reduction with the introduction of ginger extract into the reaction mixture, exhibiting a dependence on the dosage applied. As a result, hyperchromicity rises in the presence of ginger extract. These research findings are supported by a recent similar investigation [39] that discovered another natural product that can shield superoxide dismutase from glycation-induced structural changes. In this research study, autofluorescence was used to explore the detection of AGEs as a result of HSA glycation. It has been shown that the glycation of HSA results in a spectrum that is a highly distinctive feature of AGEs [44]. This could be connected to modifications brought on by glycation in the HSA molecules’ tryptophan microenvironment. In accordance with this investigation, increased concentrations of ginger extract prevented changes in the tryptophan microenvironment, exposing the tryptophan residue to a polar environment. As a consequence, ginger extract seems to guard against AGE synthesis. The biophysical results regarding the safeguarding of glycated samples by ginger extract align with the previously published literature, wherein natural products safeguarded HSA against structural alterations induced by glycation and the formation of AGEs [24,45,46]. These results demonstrate a dose-dependent inhibition of the formation of AGEs, carbonyl compounds, and ketoamines, which are products of the glycation process. Ginger extract was added to the glycation reactions of HSA molecules to prevent their structural alterations. Polyphenolic components in ginger extract may be responsible for its anti-glycating and anti-AGE properties.

Protein structural disruption is caused by a variety of chemical agents, with non-enzymatic glycation of protein molecules being one of the contributing factors. Evidence suggests that the glycation of serum albumin via various reducing sugars in the blood is an inevitable process. The in vivo homotypic aggregation (self-aggregation) occurs due to exposure of hydrophobic/inner core residues of the globular proteins that are normally present inside the protein molecules. Ginger extract showed significant inhibition in the browning intensity of glycated samples at concentrations of 50 µg/mL (26%; *p* < 0.01) and 100 µg/mL (43%; *p* < 0.001) as compared to the glycated sample without extract.

Ginger has been used for centuries both as a therapeutic agent and as a common spice in food preparation on a daily basis [14,15,16]. However, the extract from ginger was tested for its direct effect on human PBMCs. The results from the MTT assay clearly indicated that no toxic effects were observed when the ginger extract was mixed with human PBMCs. Thus, ginger extract is well accepted by the human physiological system without any side effects.

Ginger is a medicinal plant and has several therapeutic properties. Its antibacterial properties have been evaluated and established by several researchers [11,14,15,16,47], which align with the results of this study as well, demonstrating that ginger extract exhibits moderate to potent antibacterial activity. A more pronounced inhibitory function was observed against Gram-positive bacteria compared to Gram-negative bacteria. The findings revealed that the *B. subtilis* standard strain was the most susceptible to the extract.

It is well known that HSA is one of the most abundant proteins (35–50 g/L) in the human circulatory system [48]. Most of the HSA presents as non-glycosylated protein [49] in the system and serves several important physiological functions such as restoration of blood volume, shock treatment, acute management of burns, and antioxidant functions [49,50]. HSA possesses a unique ability of binding with a variety of molecules like hormones, bilirubin, fatty acids, metal ions, and several kinds of drugs [49,51] and serves as an important pharmaceutical vehicle. Binding properties and clinical functions of HSA are highly dependent on its structure and conformation. We have shown in spectral studies that glycation of HSA results in conformational alterations. In CD analysis, it has been further confirmed that secondary structure changes also occurred due to glycation. Native HSA has a higher percent of α-helix structure; however, the glycation process induces the β-sheet structure in HSA. CD analysis revealed changes in the secondary structure of glycated samples, both with or without ginger extract. The addition of ginger extract (100 µg/mL) exhibited a significant inhibition or reduction in both β- and α-secondary structures changes, thereby protecting HSA proteins from substantial conformational modifications induced by glycation. The therapeutic potential and clinical benefits of ginger extract should be further evaluated in the prevention of chronic diseases including diabetes and associated complications.

Based on the previous published literature, compounds (α-curcumene, β-sesquiphellandrene, zingiberene, gingerol, 8-shogaol, α-sitosterol, and β-bisabolene) [17,18,19,20] that are potentially present in the aqueous extract of ginger, as given in Table 3, were studied in in silico molecular docking studies. The results from this study identified gingerol and 8-shogaol as the possible phytochemicals responsible for the antiglycation activities. Among the studied compounds, only compounds 4 and 5 could form an H-bond with the receptor, in addition to the other hydrophobic and van der Walls interactions. MD simulations studies, which assist in understanding the interplay between ligands and protein conformation, indicated that both complexes display lower RMSD values than the native HSA. Moreover, both complexes exhibited lower average RMSF values than the native HSA protein. Overall, gingerol and 8-shogaol targeted the active pocket of the HSA protein, providing stability and potentially protecting HSA structure and function.

**Scheme 1:**
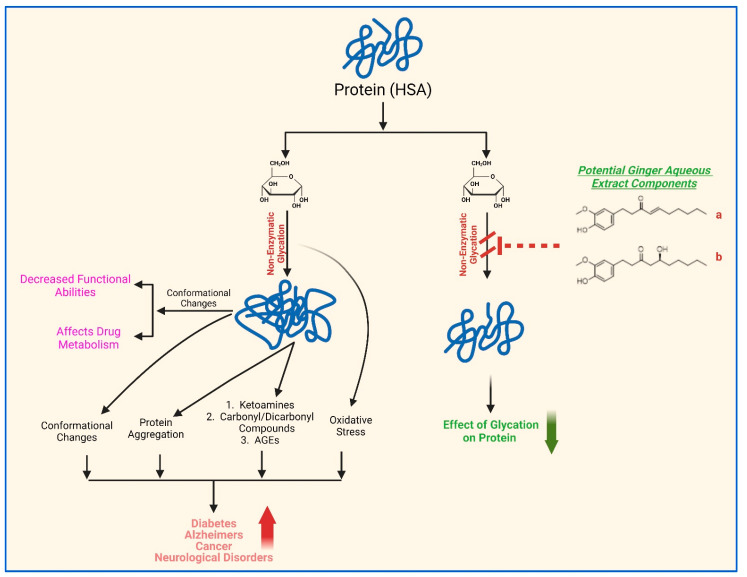
Schematic presentation of the potential role of aqueous extract of ginger in maintaining the structural integrity and function restoration of HSA. Gingerol (a) and 8-shogaol (b) are potential key components of ginger aqueous extract that act as anti-glycation agents.

Overall, these findings suggest that blood proteins can undergo non-enzymatic glycation, which may potentially be exacerbated under several abnormal physiological conditions such as hyperglycemia, stress, depression, chronic diseases, aging, etc. The glycation process induces conformational changes and aggregation of proteins. This effect may cause decreased functional abilities, impacting metabolism of HSA. Moreover, the accumulation of glycation by-products, including carbonyl compounds and advanced glycation end products, can contribute to the development of diseases such as diabetes, its related complications, Alzheimer’s disease, and cancer. This study provides substantial evidence for the protective effects of aqueous ginger extract on the structural integrity and functional restoration of HSA when introduced into glycation reactions with α-D-glucose (Scheme 1). Components of the aqueous ginger extract, particularly gingerol and 8-shogaol (Table 3, Figure 10 and Figure 11), likely play a significant role in mitigating the non-enzymatic glycation effects on HSA.

## 4. Materials and Methods

### 4.1. Biochemical Experiments

#### 4.1.1. Preparation of Aqueous Ginger Extract

Fresh ginger rhizomes (*Zingiber officinale* Roscoe) and dried roots (fully matured) were purchased from local stores in Hail, Saudi Arabia. The rhizomes were identified by a professor from the Department of Biology, College of Science, University of Hail, Saudi Arabia. The aqueous extract of ginger was prepared as described by Elosta et al. [52]. Briefly, 50 g of ginger was homogenized in 75 mL of NaCl (0.9%) at room temperature. For homogenization, the blender was set at its maximum setting, i.e., 1 min torrents for 12 min. The standardized mixture was sieved via cheesecloth before being centrifuged for 10 min at 2000× *g*. With double distilled water, a clear supernatant of up to 100 mL was obtained. All samples were prepared and kept as aliquots at −20 °C for later use. The total amount of ginger utilized, and the final volume of the extract, aided the estimation of the concentration of the aqueous ginger extract. After the extraction, 500 mg of ginger material was present in 1 mL solvent. Material confirmatory assays for phenolic chemicals and flavonoids were used as given below.

#### 4.1.2. Total Phenol Content

According to a previously published article, a test employing Folin–Ciocalteu reagent was performed to assess the total phenol content in ginger extract [45]. As a reference, various concentrations of gallic acid (50, 75, 100, 125, 150, 200, and 250 µg/mL) were used in this regard. The total phenolic concentration was calculated using the calibration plot and was shown in µg gallic acid equivalents (GAEs). Experimental examinations were conducted in triplicate. The results were given in milligram of gallic acid equivalents per gram of dried taster extract.

Total phenolic content (TPC) = X × Y/Z

where ‘X’ stands for the gallic acid concentration in mg/mL acquired from gallic acid calibration curve, ‘Y’ stands for plant extract volume in mL, and ‘Z’ represents pure plant extract weight in gram (g).

#### 4.1.3. Total Flavonoid Content

As mentioned in earlier papers, the TFC of ginger extract was estimated via the aluminum chloride (AlCl_3_) assay [45,53]. The calibration curve was constructed using various concentrations of quercetin (20–250 µg/mL). Ginger extract (500 µL, 50 µg/mL) or normal quercetin solution (various concentrations, 500 µL) were added in a tube containing 500 µL of AlCl_3_ (2%). The tubes with solutions were incubated for 1 h while being shaken intermittently. At 430 nm, a spectrophotometer measured the absorbance of each reaction mixture contrary to the blank (only ethanol). Since the 2% AlCl_3_ solution was made in ethanol, the blank was ethanol. The quercetin equivalent (mg/g) (mg QUE/g) was used to quantify the total flavonoid content.

Total flavonoid content (TFC) = X × Y/Z

where X represents the quercetin concentration (mg/mL), Y represents the sample volume (mL) consumed in the extraction, and Z signifies the weight of the pure ginger sample utilized (g).

#### 4.1.4. Hydrogen Peroxide Radical Scavenging

The capacity of ginger extract (0.78–100 µg/mL) to scavenge hydrogen peroxide was assessed using a recently reported procedure [53]. A UV–visible spectrophotometer was used to evaluate the absorbance of H_2_O_2_ versus phosphate buffer free of H_2_O_2_ (blank) at a persistent wavelength (230 nm). The naturally occurring organic compound ‘ascorbic acid’ has antioxidant properties as well as a redox buffering function, decreasing/neutralizing reactive oxygen species. As a result, ascorbic acid was employed as a control antioxidant in this study. The experiment was carried out 3 times. The succeeding calculation was used to calculate the proportion of scavenged H_2_O_2_:

Percentage of scavenged H_2_O_2_ = [(X – Y)/X] × 100

where X is the absorbance in the absence of extract (or control), and Y is the absorbance of samples having extract or control.

#### 4.1.5. Glycation of HSA by α-D-Glucose

Minor changes were made to the previously reported glycation of HSA procedure [7,54]. Briefly, the standardization of non-enzymatic glycation of HSA was first carried out. HSA samples (10 mg/mL) were prepared using 20 mM phosphate-buffered saline (PBS) (pH 7.4). Glycation was standardized using various (0.01, 0.025, 0.05, and 0.1 M) α-D-glucose concentrations. The reaction mixtures of HSA and α-D-glucose were incubated for varying time periods, i.e., 1 week (G-HSA-1W), 5 weeks (G-HSA-5W), 10 weeks (G-HSA-10W), and 15 weeks (H-HSA-15W), to analyze non-enzymatic glycation of the HSA protein. The antiglycation activity of ginger extract was verified by adding varying concentrations (0.78–100 µg/mL) of the extract to the glycation reaction. For sterility, filtration of all reaction mixtures was carried out through a 0.22 µm Millipore filter into pre-autoclaved sealed vials. HSA solutions lacking glucose were maintained alongside the experimental samples under identical experimental conditions and served as controls. Post incubation, the solutions underwent thorough dialysis against PBS (20 mM; pH 7.4) to eliminate unbound glucose. After dialysis, the concentration of protein samples was calculated by applying the Beer Lambert Law equation (as given below) using a nanodrop device (2000 UV/Vis Spectrophotometer; Thermo Scientific, Wilmington, DE, USA) [55]. Samples were preserved at −20 °C. Various quantities (0.78–100 µg/mL) of the ginger extract were investigated for their effectiveness in glycation inhibition. Various quantities of ginger extract were introduced into the reaction mixture at the same time as α-D-glucose.

$$\mathrm{Protein}\;\mathrm{concentration}=\frac{A_{\lambda \max}\times \mathrm{Mw}\times \mathrm{df}}{{\varepsilon^{1\%}}_{280\mathrm{nm}}\times l}$$

Here, A_λmax_ represents the absorbance at 280 nm, Mw is the molecular weight of HSA, df is the dilution factor, *l* is the path length of the cell (1 cm), and ε^1%^_280nm_ is the extinction coefficient of the HSA.

#### 4.1.6. Detection of Ketoamines

Using a previously established colorimetric technique that utilized NBT with minimal adjustments, the glycation of HSA samples was assessed [7,56]. A standard glycated albumin was prepared. Bovine serum albumin (BSA) (150.5 µM) was incubated for 15 days with 0.5 M glucose at room temperature in 20 mM PBS and exhibited complete glycation with subsequent production of ketoamines. Then, 50 µL of each of the samples of native HSA, glycated HSA (1, 5, 10, and 15 weeks), and glycated HSA with varying concentrations of aqueous ginger extract (0.78–100 µg/mL) was dispensed into the wells of microtiter plates in triplicate. A 100 µL NBT reagent (250 µM) prepared in 0.1 M carbonate buffer, pH 10.35, was added to test the samples and incubated for two hours at room temperature. A microplate reader (Accuris USA absorbance microplate Reader-MR9600-E; Edison, NJ, USA) was used to read the plate at 550 nm. Glycated BSA was used to generate a standard curve used for estimating ketoamines in glycated HSA samples. Various concentrations (0.78–100 µg/mL) of ginger extract were used to investigate its inhibition of ketoamine formation during glycation reactions.

#### 4.1.7. Determination of Protein-Bound Carbonyl Contents

The quantification of protein-bound carbonyl content in the samples was conducted through a previously documented method, with slight modifications [22]. The protein-bound carbonyl contents of N-HSA, G-HSA (1, 5, 10, 15 weeks), and G-HSA samples with varying concentrations of aqueous ginger extract (0.78–100 µg/mL) were determined. Briefly, 100 µL (0.1 mg) test samples were mixed with 200 µL of 7 mM 2,4-dinitrophenylhydrazine (DNPH) (prepared in 2 M HCl). The mixtures were kept for 1 hr at 25 °C. After incubation, 250 µL (4%) trichloroacetic acid (TCA) was added for the precipitation of 2,4-DNPH-hydrazones. Samples were centrifuged at 14,000× *g* for 5 min. The supernatant was discarded and pellets were resuspended in ethanol–ethylacetate in a ratio of 1:1 to remove unreacted DNPH. This step was repeated three times for complete removal of unreacted DNPH. The pellets were resuspended in 6 M guanidinium HCl [prepared in 20 mM PBS, pH 2.5 (adjusted by trifluoroacetic acid)]. Solutions were kept overnight at −20 °C, and the next day, solutions were thawed to facilitate complete dissolution of hydrazone. From the solutions, 100 µL aliquots were used to estimate carbonyl compounds and read at 379 nm using a microplate reader (Accuris USA absorbance microplate Reader-MR9600-E). The data were presented as the count of carbonyl groups in nanomoles per milligram of the respective protein samples. Various concentrations (0.78–100 µg/mL) of aqueous ginger extract were used to investigate the ability to inhibit the formation of protein-bound carbonyl compounds.

### 4.2. Toxicity of Ginger Extract

The MTT assay was executed following a previously outlined procedure [57], with slight adjustments, to evaluate the toxicity of the ginger extract. In summary, PBMCs at a concentration of 1 × 10^4^ cells/mL were cultured in RPMI medium, with or without extract, at various doses (0.78–100 µg/mL). PBMCs were isolated based on a previously published procedure [58]. Various samples were incubated for varying time periods at 37 °C in the presence of CO_2_ (5%) (24–72 h). The cells were then fed 100 µL of MTT (5 mg/mL). The entire medium, with the MTT solution, was aspirated from the microplate after a 4 h incubation period. The residual formazan crystals were completely solubilized in 50 µL of dimethyl sulfoxide (DMSO). Absorbance at 570 nm was subsequently measured using a microplate reader. The negative control consisted of cells that did not undergo any treatment. The percentage of cytotoxicity was determined by utilizing the background-corrected absorbance, and the calculation proceeded as follows:

(A_EXP_/A_N_) × 100 = % Cytotoxicity

where A_EXP_ stands for the experimental well’s absorbance and A_N_ for the negative control well’s absorbance.

### 4.3. Antibacterial Assay of Ginger Extract

Together with the human protein structure and function, the protective activity of ginger extract as well as the antibacterial efficacy were assessed through estimation of the minimum inhibitory concentration (MIC), employing the microdilution method, supplemented with resazurin [59]. The bacterial strains used in the assay were *Pseudomonas fluorescence* (ATCC 13525), *P. aeruginosa* (ATCC 39327), *Escherichia coli* (ATCC 11775), *Salmonella typhimurium* (ATCC 14028), *B. subtilis* (ATCC 6633), *M. tuberculosis* (ATCC 27294), and *S. aureus* (ATCC 25923). Bacterial suspensions were prepped employing the direct colony method, and the optical density of the initial suspension was adjusted through visual matching with the 0.5 McFarland standard [60]. The initial bacterial suspensions contained approximately 10^8^ colony-forming units (CFUs)/mL, and subsequent 1:100 dilutions were carried out in sterile 0.85% saline. Ginger extract was serially diluted in concentrations ranging from 20 mg/mL to 0.0012 mg/mL in 96-well plates with Mueller–Hinton broth. Each well received 10 µL of the diluted bacterial suspension, resulting in a final concentration of 5 × 10^5^ CFU/mL. Subsequently, resazurin solution (10 µL) was added to each well to indicate any microbial growth. Inoculated plates were incubated at 37 °C for 24 h. The minimum inhibitory concentration is the lowest concentration of the tested compound preventing a color change of resazurin from pink from blue. Tetracycline, dissolved in a nutrient liquid medium, served as the positive control in the experiment. A solvent control test was conducted to investigate the impact of 10% DMSO on bacterial growth. It was noted that the presence of 10% DMSO did not impede bacterial growth. Each experimental set comprised both growth control and sterility control. The tests were executed in duplicate, and the MICs remained consistent across replicates.

### 4.4. Biophysical Experiments

#### 4.4.1. Assay of AGE-Fluorophores

For the fluorometric analysis of the AGE pentosidine, samples were excited at a wavelength of 375 nm, with the peak detected between 300 and 400 nm [61]. In this experimental analysis, the slit widths for both excitation and emission were 10 nm. All protein solutions contained equal amounts of protein (60 µM).

To test if varying the concentrations of ginger extract (0.78–100 µg/mL) would prevent the formation of AGE pentosidine, the reaction mixture was applied.

#### 4.4.2. UV and Tryptophan-Specific Spectral Studies

##### UV Spectral Studies

Samples N-HSA, G-HSA-1W, G-HSA-5W, G-HSA-10W, and G-HSA-15 W as well as samples of G-HSA-10W with different concentrations (0.78–100 µg/mL) of aqueous ginger extracts were analyzed using a Shimadzu UV spectrophotometer (model UV-1700, Kyoto, Japan) and a quartz cuvette with 1 cm path length. UV absorption profiles of samples [4.5 µM (300 µg/mL); prepared in 20 mM PBS] were recorded in a wavelength range of 260 to 360 nm. A 20 mM PBS solution was used as blank. The following calculation was used to determine the percent hypochromicity at 280 nm.

Hypochromicity (%) at 280 nm = [(A_NHSA_ − A_G_)/A_NHSA_] × 100

##### Tryptophan-Specific Spectral Studies

A Hitachi model F2000 spectrofluorometer (Tokyo, Japan) was used to determine the tryptophan-specific fluorescence for all samples, i.e., the non-glycated, glycated (G-HSA-1W, G-HSA-5W, G-HSA-10W, and G-HSA-15 W), and glycated samples (G-HSA-10W) in the presence of extracts (0.78–100 µg/mL). The protein concentration used to detect AGE pentosidine in the samples was 6 µM (400 µg/mL). In order to analyze the tryptophan-specific fluorescence of the samples, a 285 nm excitation wavelength was used. The emission spectra for tryptophan fluorescence were captured between 290 and 440 nm. A slit width of 10 nm was used to analyze excitation and emission [7].

#### 4.4.3. Circular Dichroism Assay

Far-UV CD spectra of N-HSA, G-HSA-10W, and G-HSA-10W with extract (0.78–100 μg/mL) were measured using a temperature-controlled J-810 spectropolarimeter attached to an NESLAB model RYE 110 °A water bath. The spectropolarimeter was adjusted to a speed of 20 nm min^−1^, response time of 1 s, and 25 ± 0.1 °C. For each protein solution, 3 accumulations were carried out. A sample without protein was used as a blank and subtracted from the protein solution measurements to obtain accurate results. Protein samples (2.2 μM) were run in the range of 200–280 nm and a 1 mm path length cell. Data were read as mean residual ellipticity (MRE) and expressed in deg.cm^2^.dmol^−1^;

MRE = Mwθ_obs_/(10 × n × *l* × Cp)

Here, θ_obs_ represents ellipticity in millidegrees, n denotes the number of amino acid residues of HSA, l cm is the path length of the cell, Cp signifies the protein concentration (mg/mL), and Mw is the molecular weight of HSA. The Yang equation was employed to estimate the relative percentages of the secondary structure elements [62,63].

### 4.5. Human Protein Protection by Ginger Extract

#### 4.5.1. Glycation Induced Protein Browning Levels

The intensity of protein glycation can be evaluated by analyzing the browning intensity of glycated samples of HSA. The samples were diluted according to the protocol and were analyzed by recording the absorbance of samples at 420 nm [53,64].

Protein protection percent calculation = [(Ac − As)/Ac] × 100

where, As represents the absorbance of HSA + glucose + extract; Ac represents the absorbance of HSA + glucose (control).

#### 4.5.2. Protein Misfolding or Aggregation Intensity

The potential of ginger extract to inhibit protein misfolding and aggregation was assessed by measuring the absorbance of glycated HSA samples treated with extract concentrations of 50, 75, 100, 200, 300, 400, 500, and 600 µg/mL [53,64]. The protein misfolding intensity was derived using the following formula:

Percentage of protein misfolding = [A_340_/(A_280_ − A_340_)] × 100

where A_340_ = Absorbance at 340 nm and A_280_ = Absorbance at 280 nm.

### 4.6. Binding Site Definition

Autodock Vina was employed to define the binding site using rectangular boxes. The binding site, or active site, was identified based on the Uniprot database of HSA protein (P02768) [65]. To ensure accuracy, we cross-verified the binding site using the CASTp 3.0 server. Within the plugin, users have the option to designate the box center either by supplying explicit coordinates or, for user convenience, by delineating a PyMOL selection (PyMOL Version 3.0) [66,67].

#### 4.6.1. Preparation of Protein and Ligand Structures

Seven compounds were obtained from the PubChem database in the initial SDF file format [68]. These compounds were imported into the Autodock visualization panel to generate 3D ligands [69]. The 2D conformations of each molecule were transformed into high-quality 3D structures, featuring distinct conformations. This process is particularly suitable for preparing molecules for subsequent calculations involving variable bond torsions, such as docking. Subsequently, the resulting 3D compounds were individually converted into PDBQT files [70,71].

Preparation of the receptor structure involved retrieving the 3D structure file of human serum albumin from the RCSB PDB database with the PDB code 7ov1 and a resolution of 1.90 Å [72]. This structure was processed using AutoDockTools (ADT version 1.5.7). The preparation steps included the removal of water and solvent molecules, eliminating the bound ligand, adding polar hydrogens, and assignment of partial charges. The resulting modified structure was then saved in the AutoDock PDBQT format. Simultaneously, the co-crystallized ligand from the 7ov1 structure was extracted, subjected to preparation procedures, and saved in the PDBQT format using ADT for subsequent use in virtual screening [73].

Virtual screening was conducted through docking simulations utilizing AutoDock Vina. The search space center, determined as (24.2, −3.514, 16.872), focused on the binding pocket, with dimensions set to 110, 110, and 114 °A to encompass the active site of HSA. Default AutoDock Vina parameters were maintained during the docking process, constituting a virtual screening experiment. The configuration and implementation of docking runs utilized the PDBQT file format, an adapted version of the protein data bank format that incorporates atomic charges, atom type definitions, and topological information specific to ligands, including details on rotatable bonds. The preparation of these files was facilitated by the plugin, employing scripts from the Autodock Tools package. Ligands for subsequent docking runs could be prepared either individually through PyMOL selections or by specifying a directory containing a library of ligands, earmarked for docking [74].

For binding site analysis employing interaction maps, AutoDock utilized autogrid to calculate these maps before the actual docking run. Interaction energies between ligand atoms and the receptor were computed across the discretized grid representing the binding site. This approach streamlined the docking process by avoiding the need to calculate interaction energies at each step, and the grid maps also offered insights for ligand optimization. Visual inspection of the grid maps could reveal potential areas with a presence of unsaturated hydrogen acceptors or donors and unfavorable spatial overlaps between the ligand and the receptor.

#### 4.6.2. Molecular Dynamics Simulation

The methodology employed for the MD simulation study, utilizing the Desmond Schrödinger software (Maestro, trial version of Schrödinger software, LLC, NY, USA), aimed to investigate conformational changes in the protein induced by the ligand binding site. Additionally, it aimed to evaluate the influence of these alterations on the protein–ligand complex. To assess the interaction of the HSA protein molecule with the two most promising ligand molecules, as well as stability, a simulation study was conducted over a 100 ns period using Desmond v12. The initial step involved placing the complex in a protein preparation wizard for optimization, analysis, and refinement, utilizing the docking complex system builder menu setup.

Incorporation of water molecules into the HSA protein docking complex employed the simple point charge (SPC) water model. The system builder was constructed with counter ions, implementing the shake algorithm to constrain water molecule geometry. The bond lengths involving heavy atoms and hydrogen were preserved, and electrostatic interactions were implemented utilizing the Particle Mesh Ewald (PME) method. Orthorhombic periodic boundary conditions (PBCs) were established. Energy minimization was performed employing a maximum of 5000 steps utilizing the steepest descent algorithm (SD) and 1000 steps utilizing the conjugate-gradient algorithm (CG), with a convergence threshold set at 50 KJ/mol. The subsequent dynamics simulation spanned 100 ns, during which the length of hydrogen-involved bonds was constrained in an NPT ensemble without restraints. A brief simulation time of 1.2 picoseconds (ps) at a temperature of 300 K was used to relax the system.

### 4.7. Statistical Analysis

The mean and standard deviation (mean ± SD) of the entire dataset were presented. Student’s *t*-test and one-way analysis of variance (ANOVA) with GraphPad Prism 4.0 software for Windows 10 (Boston, MA, USA) was performed. *p* values < 0.05 were considered significant. Each sample was run in triplicate.

## 5. Conclusions

Glycation interactions with biomolecules result in the formation of highly toxic biochemical compounds that can adversely affect human health directly or indirectly. Natural products have demonstrated several health advantages, with ginger being prominently recognized for its therapeutic properties. It has been demonstrated that ginger and its active ingredients are crucial in managing various diseases. Furthermore, ginger extract can decrease the glycation burden on blood proteins. This research study has demonstrated that aqueous ginger extract components exhibit strong antioxidant, anti-glycating, and anti-AGE activities. As a consequence of these findings, ginger extract may serve as an alternative therapeutic agent for diabetes and other chronic complications. However, further in-depth and long-term in vivo investigations in animal models are necessary to validate these findings and elucidate the underlying mechanisms of the phytoconstituents in aqueous ginger extracts. Additionally, dose-dependent clinical trials in patients are essential to assess safety and mitigate the risk of toxicity or adverse reactions.

## Figures and Tables

**Table 1:** Antibacterial activity of aqueous extract of ginger.

		Minimum Inhibition Concentration (mg/mL)	
Bacterial Strains	Ginger Extract	Ginger Extract + HSA	Tetracycline
*P. fluorescence*	10.30	9.10	1.11
*P. aeruginosa*	21.00	16.44 *	7.20
*E. coli*	13.61	12.19	1.68
*S. typhimurium*	29.00	27.00	1.87
*B. subtilis*	0.39	0.34 *	1.61
*M. tuberculosis*	20.20	17.30 *	0.97
*S.* *aureus*	41.00	43.00	1.94

* Minimum inhibitory concentration (MIC) values are given as mg/mL for extract and µg/mL for tetracycline.

**Table 2:** Secondary structure analysis of N-HSA, G-HSA-10W and G-HSA-10W with inhibitors (varying concentrations of ginger extract).

Conformation	N-HSA	G-HSA-10W + Extract (µg/mL)									
		0	0.78	1.56	3.12	6.25	12.5	25	50	100	AG
α-helix	44.6 ± 0.18	35.7 ± 0.15 ** (−19.96%)	35.6 ± 0.16 ** (−20.18%)	35.8 ± 0.14 ** (−19.73%)	35.6 ± 0.15 ** (−20.18%)	35.5 ± 0.19 ** (−20.40%)	36.3 ± 0.16 ** (−18.61%)	36.6 ± 0.19 ** (−17.94%)	38.6 ± 0.15 * (−13.45%)	41.9 ± 0.14 (−6.05%)	41.1 ± 0.17 (−7.85%)
β-sheet	26.4 ± 0.16	33.2 ± 0.17 *** (+25.78%)	33.3 ± 0.18 *** (+26.14%)	33.1 ± 0.16 *** (+25.38%)	33.3 ± 0.16 *** (+26.14%)	33.3 ± 0.17 *** (+26.14%)	32.3 ± 0.18 *** (+22.35%)	32.2 ± 0.16 ** (+21.97%)	30.6 ± 0.18 ** (+15.90%)	27.5 ± 0.17 (+4.17%)	27.9 ± 0.15 (+5.68%)
β-turn	19.5 ± 0.19	20.5 ± 0.18 (+5.13%)	20.5 ± 0.13 (+5.13%)	20.5 ± 0.18 (+5.13%)	20.6 ± 0.14 (+5.64%)	20.5 ± 0.15 (+5.13%)	20.5 ± 0.14 (+5.13%)	20.4 ± 0.12 (+4.62%)	20.0 ± 0.12 (+2.56%)	19.5 ± 0.10 (0%)	19.8 ± 0.12 (+1.54%)
Random coil	11.5 ± 0.13	10.9 ± 0.14 (−5.21%)	10.9 ± 0.15 (−5.21%)	10.9 ± 0.16 (−5.21%)	11.0 ± 0.13 (−4.35%)	11 ± 0.12 (−4.35%)	10.9 ± 0.11 (−5.21%)	10.9 ± 0.13 (−5.21%)	11.0 ± 0.11 (−4.35%)	11.1 ± 0.09 (−3.48%)	11.2 ± 0.10 (−2.61%)

Data are given as a percentage of the secondary structure elements. Samples run in triplicate and values are mean ± standard deviation. Significant difference is given as *, **, and *** representing *p* < 0.05, *p* < 0.01, and *p* < 0.001, respectively, versus N-HSA. Values in parentheses represent the percent change in the secondary structure of N-HSA. Percent decrease and increase are denoted by “–” and “+” signs. Varying concentrations of ginger extract (0–100 μg/mL) were used in the glycation reaction as inhibitors. *t* test was adopted for the comparison between the groups. Aminoguanidine (AG), a potent glycation inhibitor, was used as positive control.

**Table 3:** Docking score of compounds **1**–**7** along with the H-bond, RMSD, and RMSF values.

Comp. No.	Compounds	Binding Affinity (kcal/mol)	H-Bonds	Average RMSD	Average RMSF
**1**	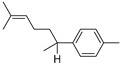 α-Curcumene	−6.9	-	-	-
**2**	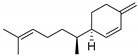 β-Sesquiphellandrene	−6.7	-	-	-
**3**	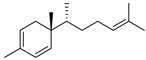 Zingiberen	−6.1	-	-	-
**4**	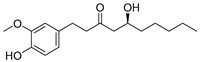 Gingerol	−7.4	Leu46 and Leu90	3.53	1.42
**5**	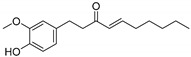 8-Shogaol	−7.7	Arg34	3.28	1.47
**6**	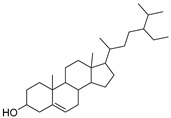 α-Sitosterol	−6.1	Asn135 and Leu136	-	-
**7**	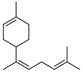 α-Bisabolene	−6.8	-	-	-

## Data Availability

The original contributions presented in the study are included in the article, further inquiries can be directed to the corresponding authors.
